# The Influence of Various Chemical Modifications of Sheep Wool Fibers on the Long-Term Mechanical Properties of Sheep Wool/PLA Biocomposites

**DOI:** 10.3390/ma18133056

**Published:** 2025-06-27

**Authors:** Piotr Szatkowski

**Affiliations:** Department of Glass Technology and Amorphous Coatings, Faculty of Materials Science and Ceramics, AGH University of Krakow, Al. Mickiewicza 30, 30-059 Krakow, Poland; pszatko@agh.edu.pl

**Keywords:** sheep wool fibers, biocomposites, natural

## Abstract

Sheep wool is a natural fiber from various sheep breeds, mainly used in clothing for its insulation properties. It makes up a small share of global fiber production, which is declining as synthetic fibers replace wool and meat farming becomes more profitable. Wool from slaughter sheep, often unsuitable for textiles, is treated as biodegradable waste. The aim of the study was to develop a fully biodegradable composite of natural origin from a polylactide (PLA) matrix reinforced with sheep wool and to select the optimal modifications (chemical) of sheep wool fibers to obtain modified properties, including mechanical properties. The behavior of the composites after exposure to aging conditions simulating naturally occurring stimuli causing biodegradation and thus changes in the material’s performance over its lifespan was also examined. Dynamic thermal analysis was used to describe and parameterize the obtained data and their variables, and the mechanical properties were investigated. The research culminated in a microscopic analysis along with changes in surface properties. The study demonstrated that wool-reinforced composites exhibited significantly improved resistance to UV degradation compared to pure PLA, with samples containing 15% unmodified wool showing a 54% increase in storage modulus at 0 °C after aging. Chemical modifications using nitric acid, iron compounds, and tar were successfully implemented to enhance fiber–matrix compatibility, resulting in increased glass transition temperatures and modified mechanical properties. Although wool fiber is not a good choice for modifications to increase mechanical strength, adding wool fiber does not improve mechanical properties but also does not worsen them much. Wool fibers are a good filler that accelerates degradation and are also a waste, which reduces the potential costs of producing such a biocomposite. The research established that these biocomposites maintain sufficient mechanical properties for packaging applications while offering better environmental resistance than pure polylactide, contributing to the development of circular economy solutions for agricultural waste valorization. So far, no studies have been conducted in the literature on the influence of sheep wool and its modified versions on the mechanical properties and the influence of modification on the degradation rate of PLA/sheep wool biocomposites.

## 1. Introduction

Sheep wool is a natural fiber of animal origin, obtained from various breeds of sheep [1]. Until now, sheep wool has been most commonly used in the textile industry due to its biological characteristics (e.g., thermal insulation, ability to spin and felt) [2]. Sheep wool constitutes a relatively small percentage of fibers produced worldwide, accounting for no more than 3% of all fibers at the end of the 20th century [3,4]. Despite this, its natural properties make it suitable for use in composite materials for various applications in construction, agriculture, and other industries [5,6]. As a natural fiber, sheep wool exhibits thermal conductivity ranging from 0.0385 to 0.043 W/mK. This low thermal conductivity makes it an excellent material for designing composites used in construction and acoustic insulation. The thermal resistance of sheep wool is approximately 0.0065 km^2^W^−1^, further supporting its insulation capabilities (Table 1) [6,7,8].

Research on the thermal conductivity and density of various fibers has shown that felt made from sheep wool is a material that insulates heat four times better than the best-performing polypropylene fibers in this comparison [6,7]. Additionally, it demonstrates ease of adhesion to polymers. Notably, wool felt has three times lower density, resulting in thermal conductivity nine times lower than commonly used polypropylene nonwovens.

Due to the physicochemical properties of natural fibers (such as strength, elongation at break, and elastic modulus), composites reinforced with these fibers are increasingly being used in industries such as transportation (automotive and aviation), military applications, construction, and packaging (Table 2) [9,10,11].

A comparison of the properties of natural fibers, including wool, shows a wide range of strength parameters, from 120 MPa for wool to up to 900 MPa for flax fibers. When comparing these values to synthetic fibers, such as glass fiber, with a strength of 4800 MPa, they may seem very low. However, it is important to note that natural fibers, including wool, are 2.5 times lighter than glass fibers, which translates to more favorable specific strength values (i.e., the ratio of absolute strength to material density). Thus, after such a comparison of parameters, flax has a specific strength similar to that of glass fiber [12].

In the case of plant fibers, the cellulose content has the greatest impact on the strength value, especially the length of the biochemical chain and the phase in which cellulose occurs in a given fiber [13,14,15]. For wool, the high fiber strength value depends on the quality of keratin as a protein present in the middle and inner layers of the hair [8,10].

Elastic moduli in natural fibers depend mainly on mechanical strength, while a higher Young’s moduli will be observed in fibers with a higher crystalline phase content. Moreover, natural fibers do not contribute to environmental degradation and are easily disposable, even in composters [10].

The decisive condition for the use of wool fibers in composite materials is good adhesion of keratin to the applied matrix, as it determines other important properties [11]. The main purpose of wool as a modifier in composite materials is in the building materials industry. One of the attempts to use wool fibers is incorporating sheep wool into mortar and concrete. It is also being tested as a modifier in epoxy composites [16]. Wool acts as a reinforcement and contributes to maintaining the integrity of the composite. The issue of the ecological crisis, the EU’s pursuit of a closed-loop economy, and the ongoing drive to create increasingly better and more economical solutions in every area of life mean that resources allocated to this field will only grow [17,18] An important issue is the attempts to recycle and remove plastic materials from circulation, which have a very long decomposition time. However, we must not forget about the potential replacement of non-biodegradable materials made from fossil materials (such as petroleum and its refining products) [19].

A dominant trend in recent years has been research into polylactic acid (PLA) composites reinforced with wool fibers [20,21,22,23]. Of particular importance are studies on biodegradable PLA/sheep wool composites, which demonstrate improved mechanical properties and enhanced resistance to UV degradation [22]. Another significant area involves research on epoxy matrix composites reinforced with wool fibers [24,25,26]. The latest studies focus on hybrid biocomposites incorporating nanofillers such as nanosilica [24]. The growing interest in sustainable materials has led to intensive research on the use of waste wool in composites [27,28]. Applications include biodegradable packaging, building materials, and thermal insulation [27].

The aim of the conducted research was to develop a biodegradable and bio-based composite using sheep wool with properties similar to widely used polystyrene. Additionally, the study examined the impact of various chemical modifications of sheep wool fibers on the properties of biocomposites containing sheep wool and polylactide (PLA).

## 2. Materials and Methods

### 2.1. Materials and Raw Materials

For the production of composites, polylactide manufactured by NatureWorks, sold under the trade name Ingeo™ Biopolymer 3100HP (Blair, NE, USA), was used as the polymer matrix. Sheep wool was obtained from sheep breeding conducted at the Experimental Station in Bielany, belonging to the University of Agriculture in Krakow named after Hugo Kołłątaj (Cracow, Poland).

### 2.2. Preparation of Materials and Raw Materials

In the first stage of research concerning fiber preparation, the wool was cleaned (washed using natural soap), then dried at 90 °C for 5 h. Finally, it was cut into uniform fibers with a length of 3–7 mm. The prepared fibers were then subjected to modifying substances for 7 days (FeCl_2_, HNO_3_, tar).

In the second stage of research, the wool underwent modification processes where the following were applied:

1. Denaturation of wool proteins under the influence of 5% HNO_3_ (200 mL solution per 10 g of wool—xanthoproteic reaction);
2. Incorporation of iron atoms into peptide chains, under the influence of iron and its salts (FeCl_2_)—4.5 g FeCl_2_ along with 4.5 g Fe per 200 mL H_2_O;
3. Incorporation of cyclic hydrocarbons into peptide chains, under the influence of tar (wood tar)—10 g of wool immersed in a solution of 10 g tar in 200 mL methanol.

**Figure 1:**
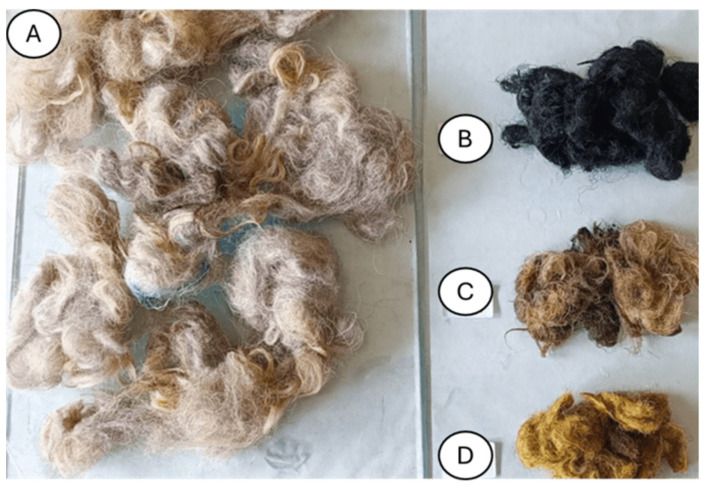
Unmodified wool (**A**) and modified wool: with tar (**B**), with iron and iron chloride (**C**), and with HNO_3_ (**D**).

The control sample was a composite of unmodified wool and pure polylactide, while the experimental samples were composites with tar, iron and iron chloride, and HNO_3_ (Figure 1).

### 2.3. Method of Producing PLA/Wool Fiber Biocomposite Samples

Samples were produced using the injection method on a Zamac Mercator (Skawina, Poland) injection molding machine (piston injection molding). The injection process consisted of several stages:

1. Modification/drying of the modifier;
2. Drying of PLA granules;
3. Compounding (mixing) of the modifier with the PLA matrix in a short twin-screw extruder L/D 25:
  Section I—PLA feed 170 °C melting PLA (water-cooled feed), Section II—170 °C (side feed of sheep wool), Section III—175 °C (mixing and homogenization), Section IV—180 °C (regranulation), with a compound speed of 100 rpm;
4. Filling the injection chamber (chamber temperature of 215 °C for pure polylactide, reduced to 180 °C for composites with wool addition) with granulate (4.0 g) (4 min of melting PLA/sheep wool mass);
5. Placing the chamber in the axis of the piston with the mold (mold temperature 80 °C) (using a mold temperature of 80 °C in PLA/sheep wool composite research is fully justified because it optimizes crystallinity, mechanical properties, and interfacial adhesion by leveraging the nucleating effect of wool fibers, in line with literature recommendations for natural fiber-reinforced PLA);
6. Starting extrusion with 10 kilo Newtons of force for 10 s;
7. Removing the finished sample from the mold.

A total of 50% of each sample from the produced composites were subjected to aging in soil under the influence of UV radiation and moisture to simulate natural conditions.

### 2.4. Types of PLA/Wool Fiber Biocomposite Samples Tested

The composites were produced using the injection method in two quantitative variants of reinforcement—5% and 15% by weight of wool (they are listed in the Table 3).

Experimental setup:

1. Control sample: pure PLA;
2. Control sample: PLA matrix/modifier unmodified sheep wool;
3. Experimental sample: PLA matrix/modifier modified wool (with tar)—5% reinforcement;
4. Experimental sample: PLA matrix/modifier modified wool (with tar)—15% reinforcement;
5. Experimental sample: PLA matrix/modifier modified wool (with iron and iron chloride)—5% reinforcement;
6. Experimental sample: PLA matrix/modifier modified wool (with iron and iron chloride)—15% reinforcement;
7. Experimental sample: PLA matrix/modifier modified wool (with HNO_3_)—5% reinforcement;
8. Experimental sample: PLA matrix/modifier modified wool (with HNO_3_)—15% reinforcement.

### 2.5. Method of Aging Biocomposite Samples

**Figure 2:**
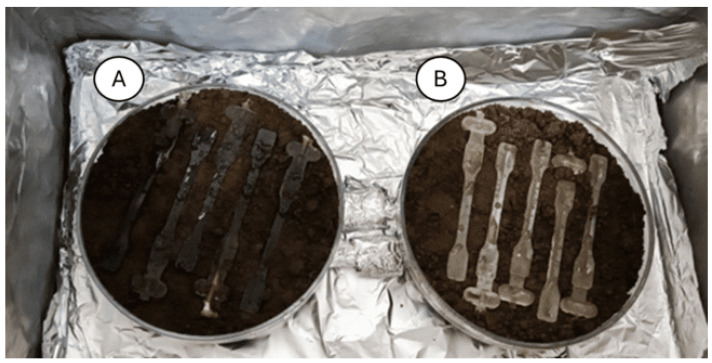
Biocomposite samples of PLA/sheep wool in soil containers during the aging process, (**A**) with PLA/sheep wool composites on the left, (**B**) pure PLA on the right.

The biocomposite samples were subjected to an aging process. This process involved placing the samples in containers filled with soil (pH 5.9) in a UV chamber (Figure 2). In the study, broad-spectrum UV radiation was used, containing both UVA (315–400 nm) and UVB (280–315 nm) components, simulating the solar spectrum. The aging process lasted for 7 days. To maintain conditions similar to those occurring when samples are exposed to sun and water, water was introduced into the containers every two days (watering).

### 2.6. Research Methods

DMA (DMA 850 Discovery of TA Instruments (New Castle, DE, USA) was used by mounting the sample on the device, but the middle part of the sample was subjected to oscillating motion with a frequency of 10 Hz and an amplitude of 10 µm. Firstly, the sample was cooled down to −40 °C and subsequently was heated up with a velocity of 5 °C/min to a temperature of 80 °C.

The tensile tests on samples were performed using a Zwick/Roell testing machine (Ulm, Germany) based on the UNE EN ISO 527-1:2020-01 standard [29]. These tests were carried out with a constant velocity of moveable traverse of 2 mm/min. As a result of stretching the samples, the plots of force vs. elongation were obtained. The dimensions of the samples were based on a standard: L = 80 mm, a1 = 15 mm, w1 = 5 mm, w = 10 mm, and mean thickness t = 4 mm. Mean weights of the samples amounted to 1.6 g (pure PLA) and 1.8–2.0 g (PLA + sheep wool). The Young’s modulus was determined by employing an extensometer with a gauge length of 25 mm.

Observations of the produced composites were conducted using an optical microscope. Images were taken at magnifications of 20×, 30×, 50×, 100×, 150×, and 200× using a Keyence VHX-900F digital microscope (Mechelen, Belgium). Surface roughness measurements were also performed.

**Figure 3:**
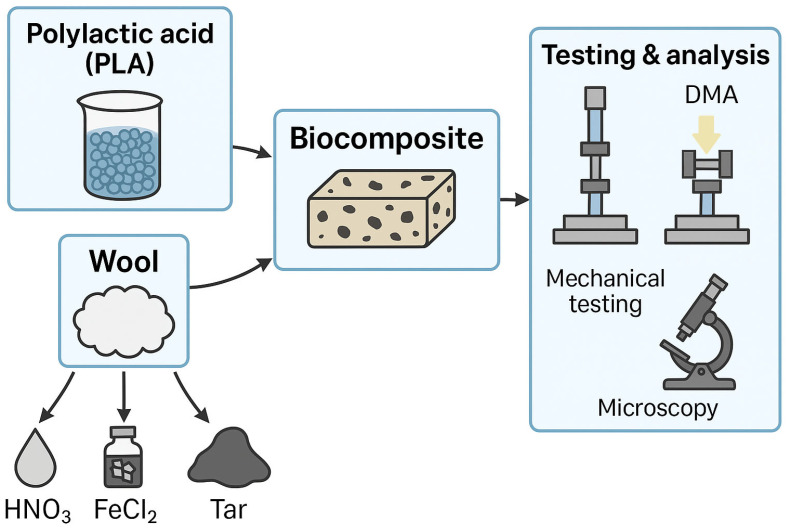
Chart of the procedure for preparing the biocomposite and modifying the PLA/sheep wool biocomposite.

For density measurements, a hydrostatic balance was used. A hydrostatic balance is an analytical balance modified to allow the weighed object to be suspended in a container of water. This method is based on Archimedes’ principle of buoyancy. Two measurements were taken, dry and wet, for each sample, and then the density of the samples was calculated using the following formula:

ρ = [m1/(m1 − m2)] × ρc (1)

where ρ is the density of the tested object, m1 is the dry mass of the object, m2 is the wet mass of the object, and ρc is the density of the liquid displaced by the immersed object. (Figure 3 shows a simplified scheme of the production of sheep wool/PLA biocomposites and the analyses of these materials).

## 3. Results

### 3.1. DMA Test Results

**Figure 4:**
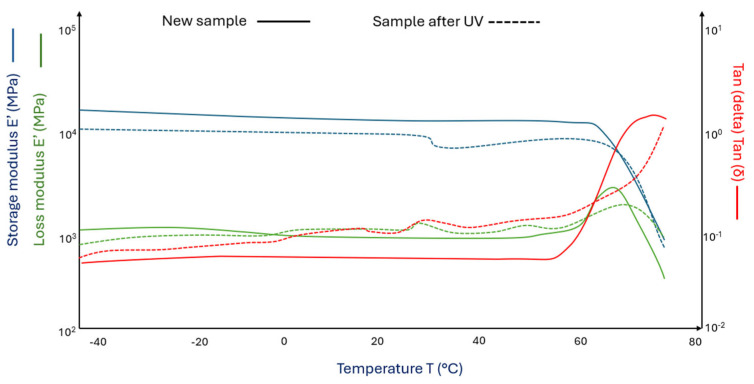
DMA graph of (^___^) pure PLA and (^_ _^) pure polylactide sample aged in a climate chamber (pure PLA after UV).

Figure 4 shows the DMA curves for pure PLA matrix samples (Figure 4 (^___^)) and PLA after the aging process in a chamber with UV + water (Figure 4 (^_ _^)).

The aging process led to significant changes in the obtained values of loss and storage moduli. PLA samples under UV influence become more susceptible to cyclic stress, with their storage modulus decreasing by 25% compared to pure PLA samples. The loss modulus in samples subjected to the aging process increases non-linearly with occurring maxima. The glass transition occurs 1 °C later in aged samples compared to the initial PLA samples. This may indicate the presence of a higher amount of crystalline phase in the aged PLA samples (radiation degradation leads to shortening of PLA chains and the possibility of chain reorganization into crystals).

**Figure 5:**
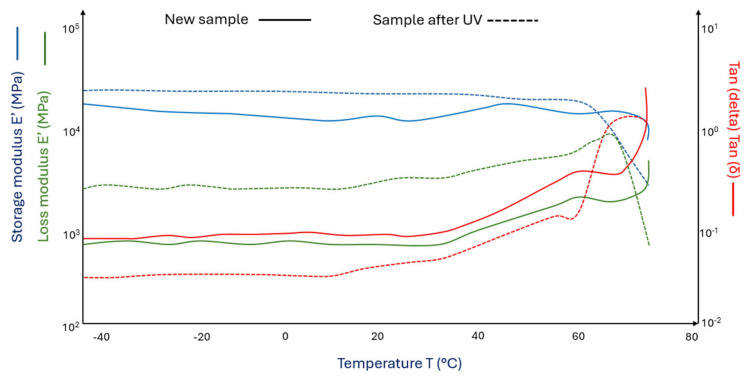
DMA graph of (^___^)PLA/pure wool 15% and (^_ _^) PLA/pure wool 15% samples after UV (aged in a climate chamber).

Figure 5 (^___^) shows the curves of the storage modulus and loss modulus for the PLA/pure wool 15% composite and after its UV aging (Figure 5 (^_ _^)).

The PLA sample modified with a 15% wool addition has a glass transition temperature almost 3 °C higher (Figure 5 (^___^)) than the pure PLA sample (Figure 4 (^___^)). However, it can be observed that the sample modified with 15% sheep wool after the aging process is characterized by a lower glass transition temperature (Figure 5 (^_ _^)) than the non-aged sample (Figure 4 (^_ _^)). In the case of PLA samples with 15% wool addition after the aging process, a significant increase in the storage modulus was observed, of 63% at 0 °C and of 9% at 50 °C (Figure 5 (^_ _^)).

**Figure 6:**
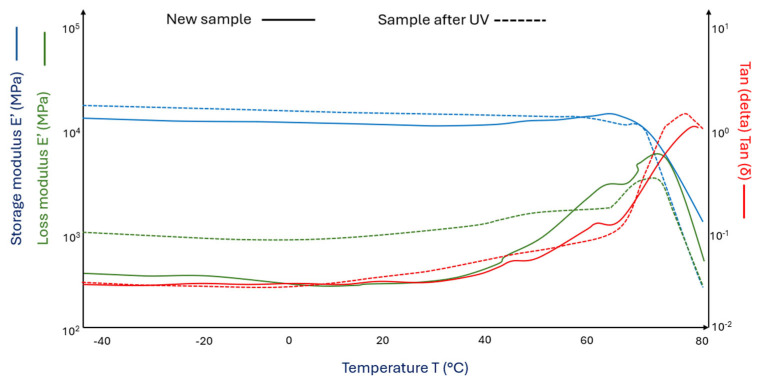
DMA graph of (^___^)PLA/acid wool 15% and (^_ _^) PLA/acid wool 15% samples after UV (aged in a climate chamber).

Figure 6 (^___^) shows the curves of the storage modulus and loss modulus for the PLA/HNO_3_-modified sheep fiber 15% composite (PLA acid wool 15%) and after its UV aging (PLA acid wool 15% after UV) (Figure 6 (^_ _^)).

In the case of PLA samples with 15% HNO_3_-modified wool addition (PLA/acid wool 15%), a decrease in the storage modulus values was observed at both 0 °C and 50 °C (Figure 6 (^___^)) compared to PLA samples modified with 15% wool without additives (Figure 5 (^___^)). However, after using nitric acid-modified wool, an increase in glass transition temperatures was observed (Figure 6 (^___^) and (^_ _^)) compared to PLA samples (Figure 4 (^___^) and (^_ _^)), as well as in PLA modified with pure wool (Figure 5 (^___^) and (^_ _^)).

**Figure 7:**
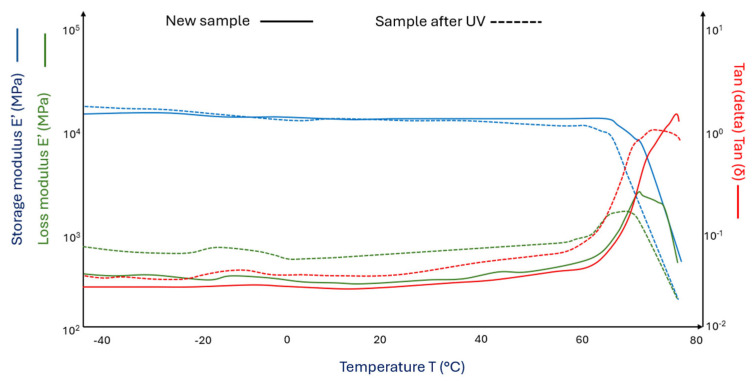
DMA graph of (^___^)PLA/Fe wool 15% and (^_ _^) PLA/Fe wool 15% samples after UV (aged in a climate chamber).

Figure 7 (^___^) shows the curves of the storage modulus and loss modulus for the PLA/Fe and Fe_2_O_3_-modified sheep fiber 15% composite (PLA/Fe wool 15%) and after its UV aging (PLA/Fe wool 15% after UV) (Figure 7 (^_ _^)).

PLA samples with iron-modified wool addition (Figure 7 (^___^) and (^_ _^)) are characterized by lower storage modulus values compared to PLA samples with unmodified wool addition (Figure 6 and 7). At 0 °C, the decrease is 14% (Figure 7 (^___^)), while at 50 °C, the storage modulus decreases by 27% (Figure 7 (^___^)). Glass transition temperatures are about 2 degrees Celsius lower (Figure 7 (^___^) and (^_ _^)) than in the case of PLA samples with 15% nitric acid-modified wool addition (Figure 6 (^___^) and (^_ _^)).

**Figure 8:**
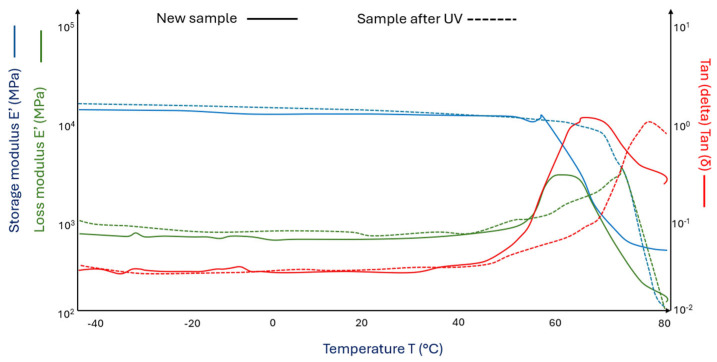
DMA graph of (^___^)PLA/tar 15% and (^_ _^) PLA/tar 15% samples after UV (aged in a climate chamber).

Figure 8 (^___^) shows the curves of the storage modulus and loss modulus for the PLA/tar-modified sheep fiber 15% composite (PLA/tar 15%) and after its UV aging (PLA/tar 15% after UV) (Figure 8 (^_ _^)).

PLA samples with 15% tar-modified wool addition (Figure 8 (^___^) and (^_ _^)) are characterized by lower storage modulus values at temperatures of 0 °C (by 5%) and 50 °C (by 20%), compared to PLA samples modified with pure wool (Figure 5 (^___^) and 5 (^_ _^)). PLA samples modified with 15% tar-added wool after the aging process (Figure 7 (^_ _^)) have higher storage modulus values, with an increase of 41% at 0 °C and 13% at 50 °C. Glass transition temperatures (Figure 8 (^___^) and (^_ _^)) are at a similar level as PLA samples with unmodified wool addition (Figure 5 (^___^) and (^_ _^)). The collected DMA results are presented in the Table 4.

After conducting the DMA study, it was found that the highest storage modulus value at 0 °C was observed in the PLA sample modified with 15% pure wool addition after the aging process (an increase of almost 54% compared to the reference PLA sample). The lowest storage modulus value at 0 °C was characteristic of the PLA sample after the aging process (a decrease of almost 22% compared to the reference PLA sample).

Considering the storage modulus value at 50 °C, the highest value was achieved by the sample modified with 15% pure wool addition after the aging process (an increase of about 35.5% compared to the reference PLA sample), while the lowest value was recorded for the PLA sample after the aging process (a decrease of almost 32% compared to the reference PLA sample). It was found that at both temperatures, 0 °C and 50 °C, the largest decreases in storage modulus were recorded in aged PLA samples without wool addition, while the largest increases were observed in samples modified with 15% pure wool addition after the aging process.

The analyzed storage modulus of the material is responsible for transferring stresses in the material volume and is responsible for its toughness. The sample modified with 15% pure wool addition after the aging process, having the highest storage modulus in the working temperature range, is characterized by the highest stiffness, while the PLA sample after the aging process with the lowest storage modulus is characterized by the lowest stiffness and toughness.

Analyzing the glass transition temperatures of individual samples, it was observed that the highest increase (about 6%) compared to the reference PLA sample was recorded for the sample modified with 15% wool with nitric acid addition after the aging process. The smallest increase in glass transition temperature was recorded for the PLA sample modified with 15% wool with tar addition. The increase in glass transition temperature of PLA samples modified with various types of wool compared to reference PLA samples is caused by the increased crystallinity of these samples and the reduced mobility of polymer chains. For this reason, a sufficient amount of energy to overcome stronger interfacial interactions increases, which causes an increase in the glass transition temperature of the samples. Dynamic Mechanical Analysis (DMA) serves as a crucial tool for evaluating viscoelastic properties of polymer composites, enabling measurement of the storage modulus, loss modulus, and glass transition temperature as functions of temperature [30]. In the case of the studied PLA/sheep wool composites, the DMA results reveal fascinating phenomena related to the influence of UV aging on mechanical properties of materials. Pure polylactide after the UV aging process exhibits characteristic signs of polymer degradation typical for thermoplastic materials. The 25% decrease in storage modulus at 0 °C and the significant reduction in mechanical properties across the entire temperature range indicates macromolecular chain scission under UV radiation influence [31].

The 1 °C upward shift in glass transition temperature (from 57 °C to 58 °C) indicates increased crystallinity of the material resulting from the reorganization of shortened polymer chains. This degradation mechanism aligns with literature data concerning PLA photodegradation, where UV radiation leads to hydrolysis and ester bond breaking [32].

**Figure 9:**
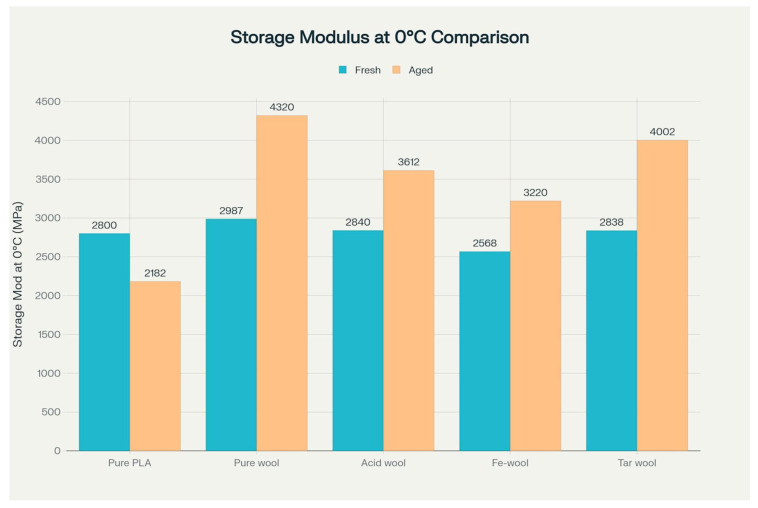
Comparison of the storage modulus at 0 °C for PLA/wool biocomposites before and after UV aging.

The most important discovery of the research is the contrary-to-expectations increase in storage modulus of all composites containing wool fibers after the UV aging process. This phenomenon represents a fundamental departure from the classical behavior of polymer materials subjected to environmental degradation [33]. Comparison of storage modulus values for all sample types tested—Figure 9.

The PLA/unmodified wool 15% composite exhibits a spectacular 54% increase in storage modulus at 0 °C after aging, significantly exceeding properties of fresh material. This unexpected effect can be attributed to the synergistic action of several mechanisms, including UV radiation blocking by fibers and induced reorganization of the matrix crystalline structure [34].

Mechanisms Responsible for Property Improvement

Wool fibers act as a physical barrier against UV radiation, limiting its penetration to deeper composite layers. This shielding effect leads to selective degradation of only surface layers while the composite core maintains structural integrity. This mechanism is particularly significant in the context of long-term durability of composites in applications exposed to solar radiation [34].

The UV aging process in the presence of wool fibers promotes the recrystallization of shortened PLA chains into ordered crystalline structures [32]. An increased crystallinity degree leads to enhanced material stiffness and improved mechanical properties, which is opposite to typical polymer degradation. Wool fibers may act as nucleation centers, facilitating crystallization and polymer chain orientation processes.

UV aging activates functional groups on keratin fiber surfaces, improving adhesion with PLA matrix. This process leads to better stress transfer between composite phases, translating to higher stiffness of the entire material. Chemical modifications of fiber surfaces during aging may create new bonds between organic phase and polymer matrix [20].

### 3.2. Results of Flexural Strength Tests

**Figure 10:**
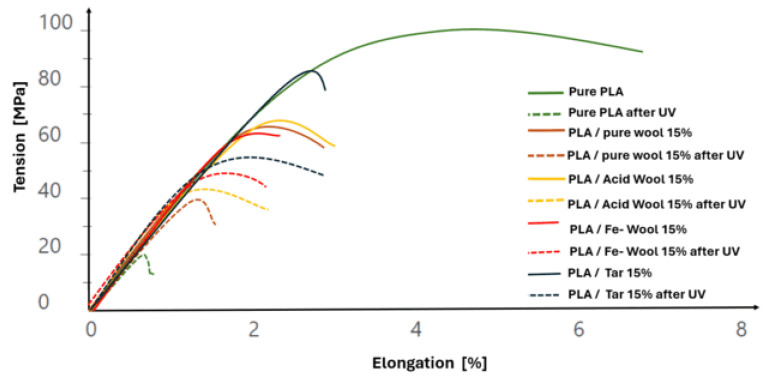
Results of the three-point bending test for PLA/wool biocomposite samples and their chemical modifications.

Modifications to wool fiber and the aging process had a significant impact on the obtained results of the Young’s modulus, mechanical strength, strain at failure, and work of fracture. These results are presented in Table 5. Additionally, Figure 10 shows example curves obtained during the flexural strength testing.

The produced composites were characterized, in most cases, by an increased Young’s modulus, with reduced strength compared to pure polylactide. Moreover, aging had a decreasing effect on the Young’s modulus of the composites. The highest strength in relation to the original samples was demonstrated by wool modified with tar, iron, and, contrary to expectations, HNO_3_ (which was expected to have lower strength compared to regular wool, due to protein degradation in the xanthoproteic reaction). Furthermore, aging resulted in higher strength than aged pure polylactide. For all composite samples, failure in the bending test occurred at a lower strain of the sample (which is characteristic for composites as materials, as they rely on precise adhesion of phases to transfer loads). Additionally, the work of destruction in each case was significantly lower than for pure polylactide, which may be due to the low strength parameters of wool, despite modifications. Mechanical analysis of PLA/sheep wool composites reveals fundamental changes in the paradigm of polymer material degradation under environmental factors. The paradoxical increase in mechanical properties after UV aging constitutes a breakthrough discovery, with broad implications for materials science. The studies confirm the suitability of these composites as biodegradable alternatives to conventional packaging materials, combining sustainability with high-performance parameters.

### 3.3. Density Results and Analysis

The measurements show that the density of the composites decreased due to the addition of wool, except for the tar-modified wool. This may be due to the presence of small air pores in the samples around the wool fibers, due to the characteristics of the manufacturing process. From a properties approach, the presence of air may be beneficial—it will lower the thermal conductivity coefficient. Density measurements show that the addition of wool reduces the density of the composites compared to pure PLA, except for tar modification. This phenomenon may result from the presence of small air pores around the wool fibers, which is characteristic of the manufacturing process. In terms of properties, the presence of air may be beneficial, as it lowers the thermal conductivity coefficient. Comparison of the obtained density values for all sample types tested on Table 6.

### 3.4. Results and Analysis of Microscopic Examinations

**Figure 11:**
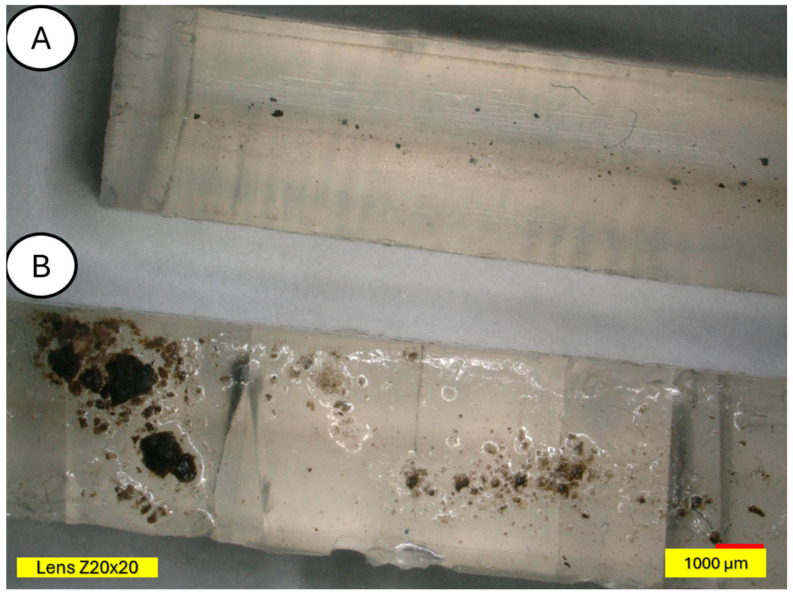
Polylactide sample (pure PLA) (**A**) and aged polylactide sample (pure PLA after UV) (**B**), magnification ×20.

**Figure 12:**
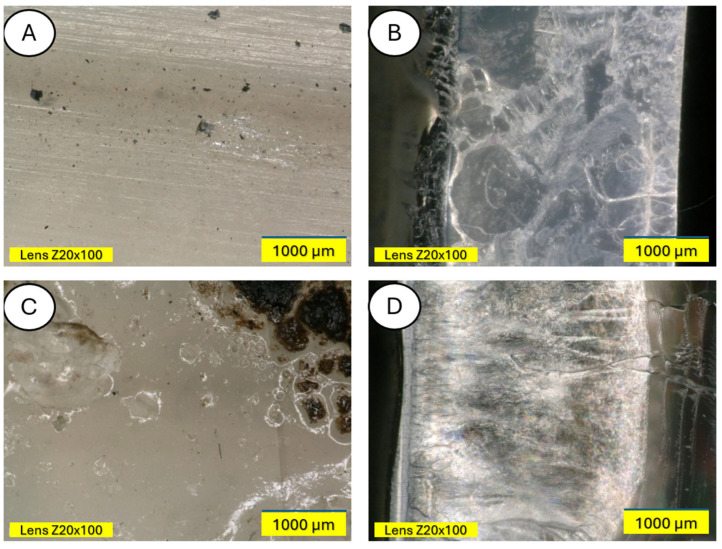
Comparison of microscopic images of polylactide samples (pure PLA) (**A**,**B**) and aged polylactide samples (pure PLA after UV) (**C**,**D**) at ×100 magnification, as well as at ×100 magnification in cross-section.

The microscopic examination was conducted on samples damaged during the three-point bending test to also obtain an image of the composite cross-section. Figure 11 and Figure 12 show a clear degradation of the reference PLA sample (A) after the aging process (B).

**Figure 13:**
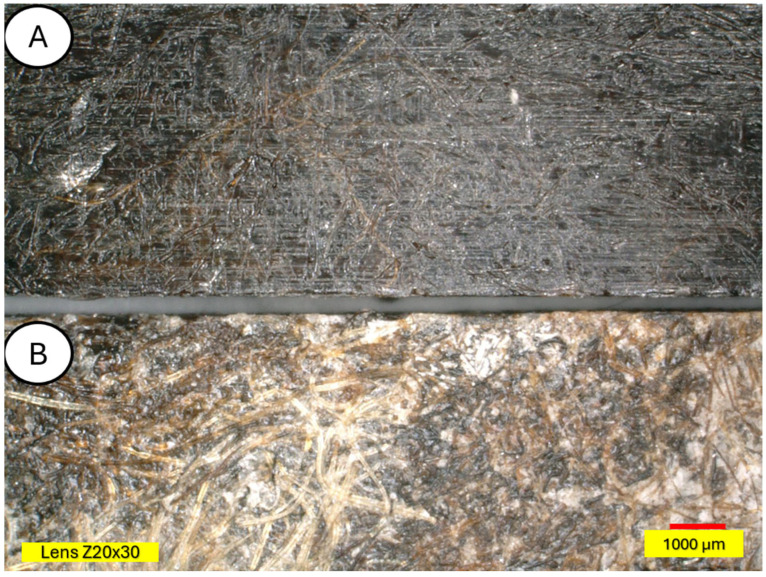
Sample with 15% wool (**A**) and aged sample with 15% wool (**B**), magnification ×30.

**Figure 14:**
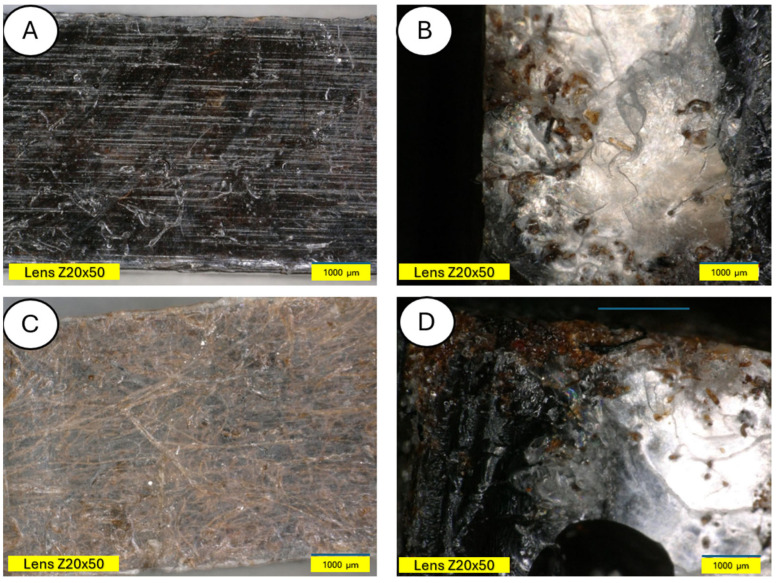
Comparison of microscopic images of composites reinforced with 15% unmodified wool (PLA/pure wool 15%) (**A**,**B**) and reinforced with 15% unmodified aged wool (PLA/pure wool 15% after UV) (**C**,**D**) at ×50 magnification, as well as at x100 magnification in the fracture plane.

There is visible increased roughness on the surface of sample B and significant mold growth. The effect of UV radiation on the PLA surface in the presence of moisture has a very significant impact on the changes occurring in the material’s structure. Pure PLA is transparent, which means that UV radiation penetration is deep and affects the entire volume of the sample. The surface of degraded PLA is wavy, indicating that there was enough energy in the system for the material, the PLA chains, to relax—to move relative to each other. Figure 13 and Figure 14 shows a PLA sample with 15% unmodified wool addition and with wool unmodified after aging.

Figure 14 shows a PLA sample with 15% unmodified wool addition (A). After aging, changes on the sample surface can be observed (B), and wool fibers have become more clearly visible. Degradation of the PLA matrix has occurred.

**Figure 15:**
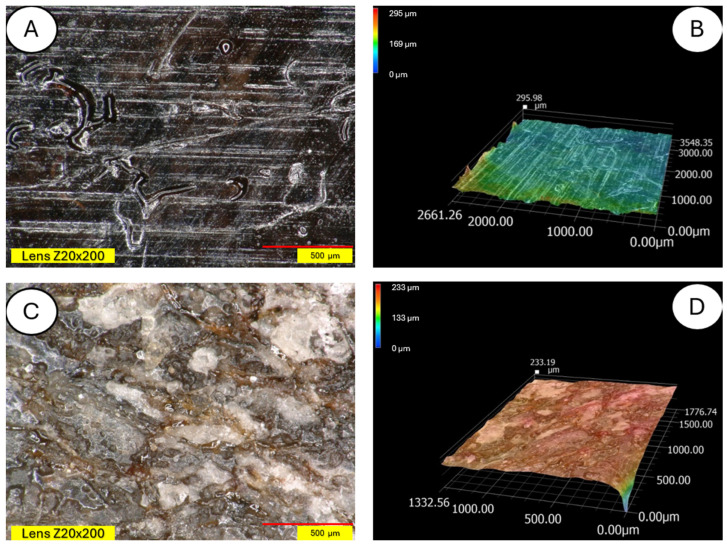
Comparison of microscopic images of composites reinforced with 15% unmodified wool and reinforced (PLA/pure wool 15%) (**A**,**B**) with 15% unmodified aged wool at ×200 magnification (PLA/pure wool 15% after UV) (**C**,**D**), as well as 3D images of its surface with color as an elevation marker made for ×200 magnification.

**Figure 16:**
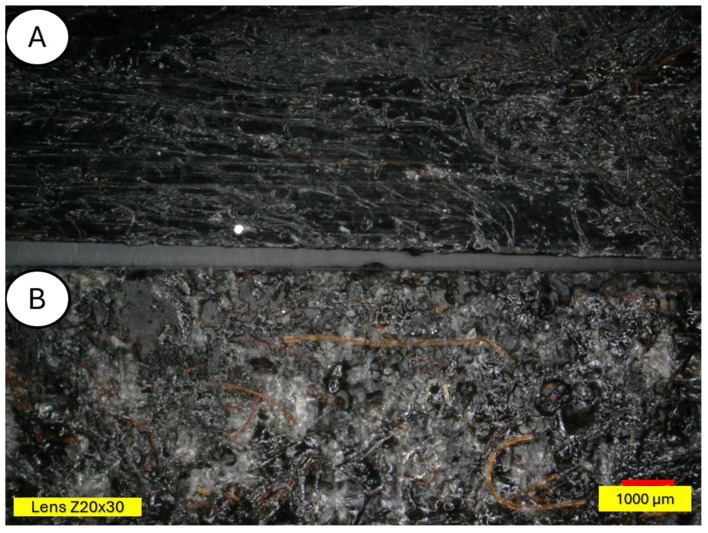
PLA sample modified with wool and nitric acid addition (PLA/acid wool 15%) (**A**) and after the aging process (PLA/acid wool 15% after UV) (**B**).

The degradation of the biocomposite containing wool fibers in its volume occurs differently than in the case of pure PLA. Wool fibers block UV radiation access to deeper parts of the samples. Visible clouding of the PLA matrix between the fibers is evident, indicating PLA recrystallization under the influence of UV rays. In Figure 15 and Figure 16, a PLA sample modified with wool and nitric acid addition can be observed. After the aging process, wool fibers and surface degradation of the PLA matrix are visible on the sample. Figure 15 shows 3D profiles of PLA biocomposites with 15% wool fiber before and after aging.

Figure 16, Figure 17, Figure 18, Figure 19, Figure 20, Figure 21, Figure 22, Figure 23 and Figure 24 show 3D profiles of PLA biocomposites with 15% wool fiber with different modifications.

Microscopic studies revealed the presence of protruding wool fiber fragments on the surface of modified samples and air pores inside the samples, in images taken in the bending plane. Moreover, surface changes resulting from aging processes are clearly visible—samples are usually lighter, and mold appears on them. All wool-modified samples changed from the original white color of polylactide to darker or completely black.

## 4. Conclusions

As part of the conducted research, efforts were made to find a substitute for polymers used mainly in the packaging industry, which is one of the largest consumers of plastics [35,36]. For this purpose, composite samples with a polylactide (PLA) matrix were prepared, which is a biodegradable and bio-derived polymer with physical properties similar to widely used polystyrene [37,38,39]. Sheep wool fiber was used as reinforcement. The wool fiber was additionally subjected to a series of chemical modifications. Namely, it was modified with nitric acid (V) (HNO_3_), iron, and tar. This was carried out to check the possibilities of modifying the composites.

The first case of using wool fibers as a reinforcing agent in cement-based composites took place in 2010. At that time, scientists investigated the potential of using clay, wool, and compounds derived from algae to produce bricks, with wool as the base [37]. For comparison, in Ref. [40], wool fiber was implemented as an additive to mortar and concrete. This resulted in a slight decrease in compressive strength. However, in the case of samples with modified wool, the compressive strength was higher than in the case of untreated fibers at all curing times. Moreover, higher tensile and flexural strength was observed for all concrete samples. Consequently, the inclusion of wool fibers in concrete also resulted in better sample continuity and higher energy absorption. Furthermore, Ref. [41] indicated that wool fibers can be successfully incorporated into matrices of different nature. It was shown that wool fibers are effective in a polymer matrix, as well as in mortar and concrete. In our own studies, it could be observed that composites using wool fiber, in most cases, showed lower strength, storage modulus, or work of fracture than pure polylactide samples. Moreover, the addition of wool caused a reduction in the decrease in these parameters under the influence of the aging process. This means that these samples, while still being biodegradable, will noticeably lose their properties more slowly compared to pure PLA, which can be beneficial. This gives the composites greater resistance to the natural environment compared to pure polylactide. Statuto et al. also showed that the inclusion of 3% (*w*/*w*) sheep wool fibers as reinforcement in clay led to a significant improvement in the mechanical properties of the material. Consequently, it was indicated that the compressive strength was significantly increased compared to unmodified adobe clay [42].

Another positive aspect occurring in composite samples relative to pure polylactide is the increase in their resistance to high temperatures. In each sample, the glass transition begins at a higher temperature than for pure polylactide (although this difference is small), and the decrease in storage modulus at high temperature is slower. The negative effects of aging, associated with the degradation of polymer chains and fungal growth, are also reduced. In each case, the samples show higher storage modulus values after aging compared to pure polylactide, which underwent the same process. For comparison, the results of an experiment conducted by Fiore et al. showed that wool fibers can be used in cement matrices to obtain mortars or plasters with improved thermal insulation properties. However, a noticeable decrease in compressive strength was also observed when adding wool fibers to the composite [43].

Microscopic examination allowed for a detailed observation of both changes in the surface of samples after aging (such as the presence of fungi) and the presence of air pores inside the samples, which may be related to the characteristics of the manufacturing process. The obtained parameters of wool-enriched composites, despite in some aspects not keeping up with the same properties in pure polylactide (such as strength), still present themselves as sufficient for applications, e.g., in the packaging industry. Another aspect of the usefulness of polylactide–wool composites in industry, which is difficult to assess, may be the reduction in production costs compared to polylactide, depending on the availability of wool on the market—sheep, even if not bred for textile purposes, need to be sheared, and the resulting wool is often wasted [9,44]. However, Ferreira et al., as well as Barone and Schmidt, noted that the mechanical strength of composites is directly proportional to the amount of fibers contained in them. It is observed that an increase in the amount of wool fibers introduced in the final product causes a decrease in the mechanical strength of the composite due to thermal abrasion during the mixing process. Moreover, the mechanical properties of polymer-based composites are significantly influenced by the degree of compatibility between the hydrophobic nature of the thermoplastic matrix and the hydrophilic nature of wool fibers. This incompatibility results in a lack of interfacial adhesion between the matrix and wool fibers. Ultimately, this can lead to a reduction in the mechanical properties of the resulting composites [45,46].

This study demonstrated that incorporating sheep wool fibers into PLA creates fully biodegradable biocomposites with improved resistance to UV-induced degradation and accelerated biodegradability compared to pure PLA. Chemical modifications of wool fibers, such as with nitric acid, iron compounds, and tar, were explored to enhance fiber–matrix compatibility and tailor mechanical and thermal properties of the composites. Among all tested compositions, the PLA composite reinforced with 15% unmodified sheep wool exhibited the highest increase in storage modulus after UV aging, indicating superior stiffness and environmental durability. These results confirm that PLA with 15% unmodified wool is the most promising composition, balancing mechanical performance, environmental resistance, and biodegradability, and making it a strong candidate for sustainable packaging applications.

## Figures and Tables

**Figure 17:**
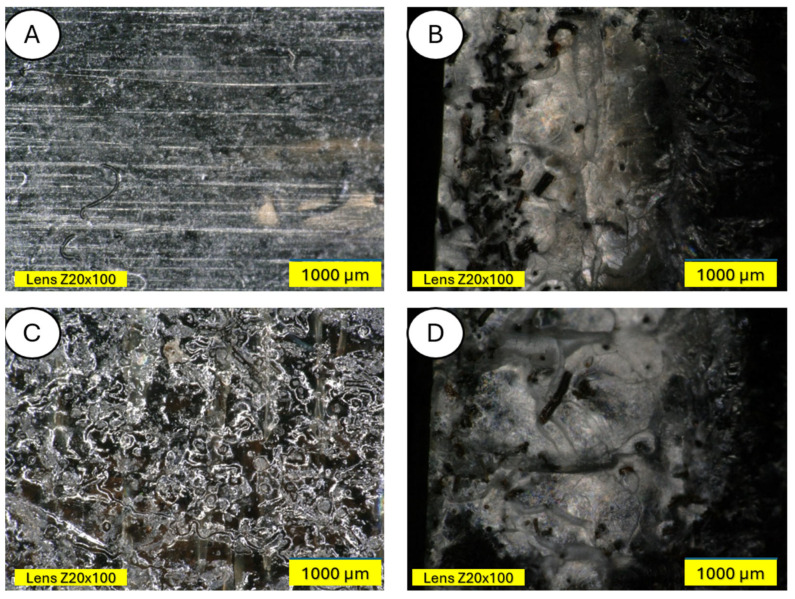
Comparison of microscopic images of composites reinforced with 15% HNO_3_-modified wool (PLA/acid Wool 15%) (**A**,**B**) and reinforced with 15% HNO_3_-modified aged wool (PLA/acid wool 15% after UV) (**C**,**D**), at ×100 magnification, as well as ×100 magnification in the fracture plane.

**Figure 18:**
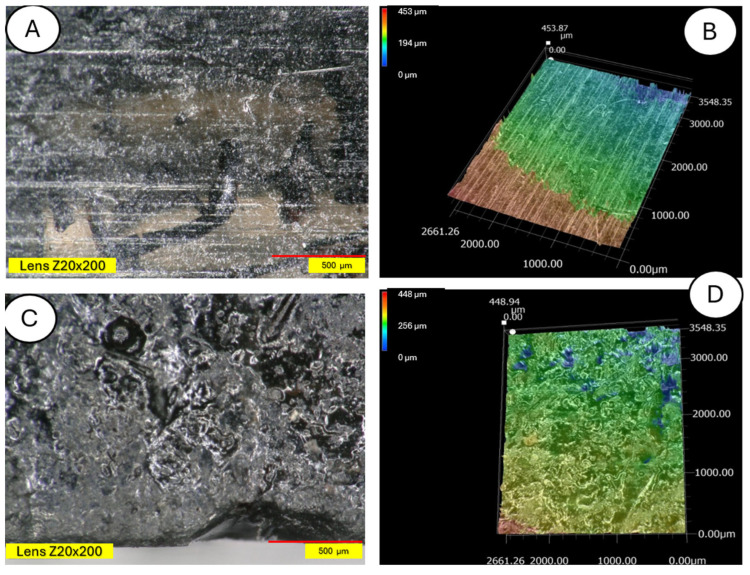
Comparison of microscopic images of composites reinforced with 15% HNO_3_-modified wool (PLA/acid wool 15) (**A**,**B**) and reinforced with 15% HNO_3_-modified aged wool at ×200 magnification (PLA/acid wool 15% after UV) (**C**,**D**), as well as 3D images of its surface with color as an elevation marker made for ×200 magnification.

**Figure 19:**
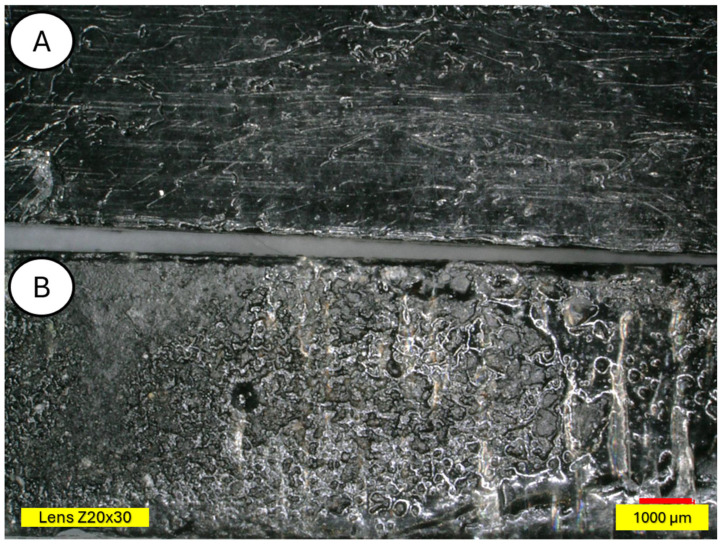
Sample of 15% iron-modified wool (PLA/Fe wool 15%) (**A**) and 15% iron-modified aged wool (PLA/Fe wool 15% after UV) (**B**), magnification ×30.

**Figure 20:**
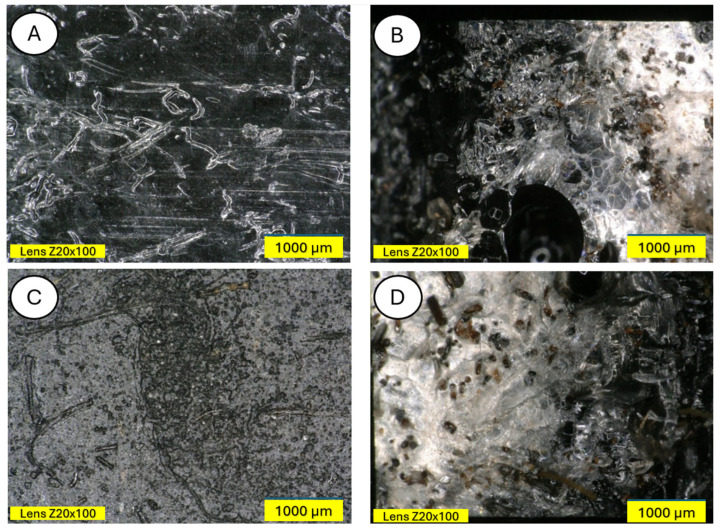
Comparison of microscopic images of composites reinforced with 15% iron-modified wool (PLA/Fe wool 15%) (**A**,**B**) and aged composites reinforced with 15% iron-modified wool (PLA/Fe wool 15% after UV) (**C**,**D**) at 100× magnification, as well as 100× magnification in the fracture plane.

**Figure 21:**
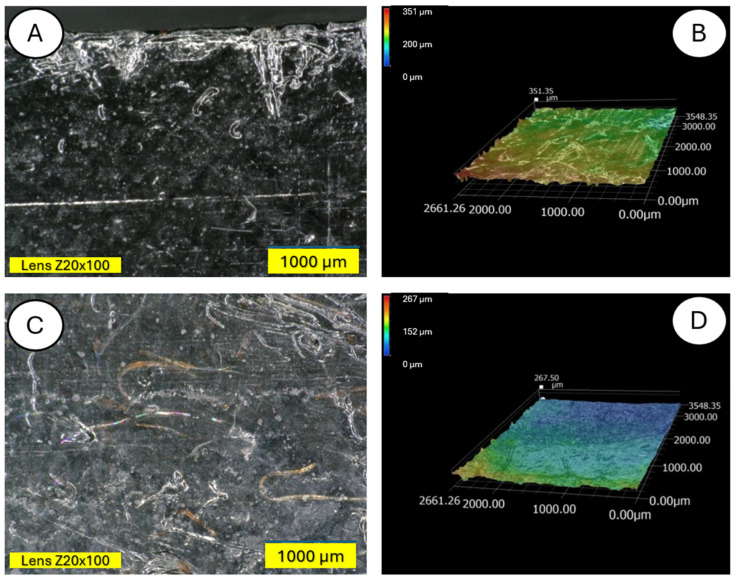
Comparison of microscopic images of composites reinforced with 15% iron-modified wool (PLA/Fe wool 15%) (**A**,**B**) and aged composites reinforced with 15% iron-modified wool (PLA/Fe wool 15% after UV) (**C**,**D**) at 100× magnification from the side of the sample, as well as 3D images of its surface with color as an elevation marker, created at 100× magnification.

**Figure 22:**
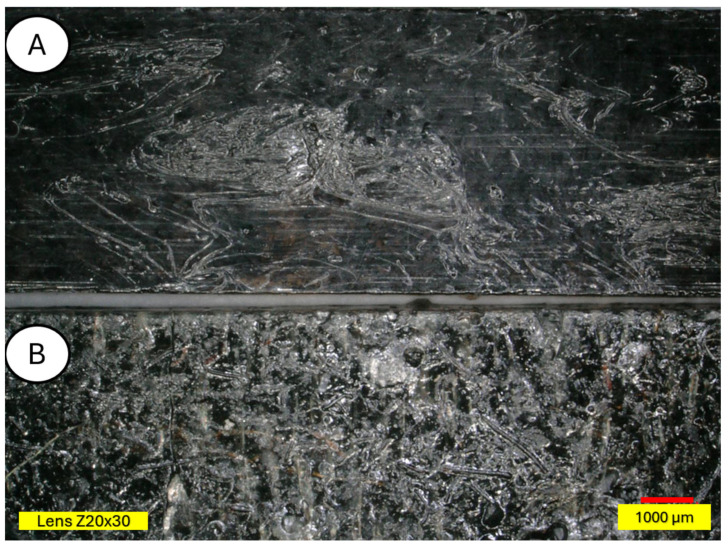
Sample of 15% tar-modified wool (PLA/tar 15%) (**A**) and 15% tar-modified aged wool (PLA/tar 15% after UV) (**B**), magnification ×30.

**Figure 23:**
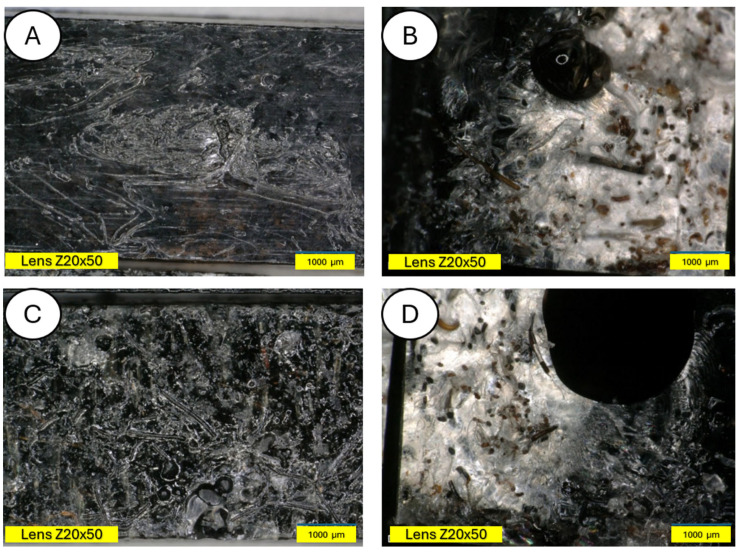
Comparison of microscopic images of composites reinforced with 15% tar-modified wool (PLA/tar 15%) (**A**,**B**) and aged composites reinforced with 15% tar-modified wool (PLA/tar 15% after UV) (**C**,**D**) at 50× magnification, as well as 100× magnification in the fracture plane.

**Figure 24:**
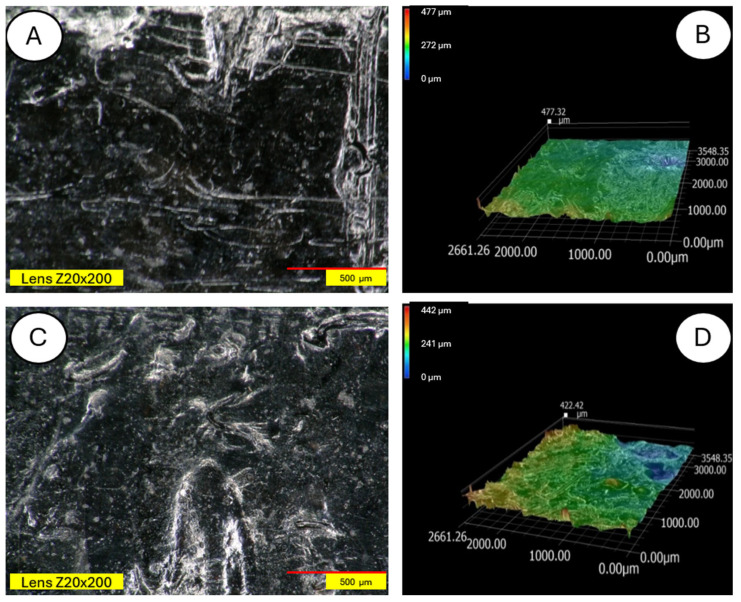
Comparison of microscopic images of composites reinforced with 15% tar-modified wool (PLA/tar 15%) (**A**,**B**) and aged composites reinforced with 15% tar-modified wool (PLA/tar 15% after UV) (**C**,**D**) at 200× magnification from the side of the sample, as well as 3D images of its surface with color as an elevation marker, created at 200× magnification.

**Table 1:** Thermal conductivity and density parameters of fibers (Andrzejewska and Topoliński 2015) [7].

Material	Density [kg m^−3^]	Thermal Conductivity [W m^−1^ K^−1^]
Polyamide 6 fiber	1140	0.25
PET fiber	1390	0.14
PP fiber	910	0.12
PE fiber	920	0.34
PVC fiber	1360	0.16
Water	100	0.58
Wool felt	320	0.047
Wool fibers	1300	0.1924
Air	1.29	0.024

**Table 2:** Comparison of physicochemical properties of natural and glass fibers (Pach, Mayer, 2010, Kim et al., 2015) [8,10].

Fibers	Strength [MPa]	Elongation at Break [%]	Elastic Modulus [MPa]
Cotton	264–654	3.0–7.0	4980–10,920
Wool	120–174	25–35	2340–3420
Silk	252–528	20–25	7320–11,220
Flax	300–900	2.7–3.2	24,000
Jute	342–672	1.7–1.8	43,800
Sisal	444–552	2.0–2.5	-
Ramie	348–816	3.6–3.8	53,400
Glass fibers	4800	1.8–3.2	86,000

**Table 3:** List of biocomposites with e-modified wool produced for testing.

Sample Name and Wool Concentration	Sample Name After Aging Process and Wool Concentration
Pure PLA	Pure PLA after UV
PLA/pure wool 5%, 15%	PLA/pure wool 5%, 15% after UV
PLA/acid wool 5%, 15%	PLA/acid wool 5%, 15% after UV
PLA/Fe wool 5%, 15%	PLA/Fe wool 5%, 15% after UV
PLA/tar 5%, 15%	PLA/tar 5%, 15% after UV

**Table 4:** DMA test results.

Sample	Glass Transition [°C]	Glass Transition Temperature Difference [%]	Storage Modulus at 0 °C [MPa]	Storage Modulus Difference at 0 °C [%]	Storage Modulus at 50 °C [MPa]	Storage Modulus Difference at 50 °C [%]
Pure PLA	60.22	-	16,212.4	-	13,460.4	-
Pure PLA after UV	61.37	1.15	12,669.7	−21.85	9191.1	−31.72
PLA/pure wool 15%	64.13	3.91	15,245.8	−5.96	16,429.3	22.06
PLA/pure wool 15% after UV	61.28	1.06	24,957.0	53.94	17,965.0	33.47
PLA/acid wool 15%	64.69	4.47	13,834.1	−14.67	15,801.9	17.40
PLA/acid wool 15% after UV	66.10	5.88	18,727.8	15.52	16,941.5	25.86
PLA/Fe wool 15%	62.12	1.90	13,397.5	−17.36	12,893.4	−4.21
PLA/Fe wool 15% after UV	63.73	3.51	15,221.1	−6.11	13,609.7	1.11
PLA/tar 15%	63.73	3.51	14,564.0	−10.17	13,698.3	1.77
PLA/tar 15% after UV	61.23	1.01	20,586.4	26.98	15,537.2	15.43

**Table 5:** Results of three-point bending for PLA/wool biocomposite samples and their chemical modifications.

Sample	Young’s Modulus [MPa]	Standard Deviation of Young’s Modulus [MPa]	Strength [MPa]	Standard Deviation of Strength [MPa]	Strain [%]	Standard Deviation of Strain [%]	Work of Destruction [N × mm]	Standard Deviation of Destruction Work [N × mm]
Pure PLA	2180	1086	97.7	2.1	7.2	0.7	781.30	91.63
Pure PLA after UV	2377	191	21.6	17.4	1.8	0.6	40.31	14.36
PLA/pure wool 15%	3717	263	38.4	31.0	1.6	1.2	78.28	102.86
PLA/pure wool 15% after UV	3030	384	39.2	19.4	2.4	0.5	102.65	50.65
PLA/acid wool 15%	3650	98	66.4	14.6	3.1	0.6	214.86	87.67
PLA/acid wool 15% after UV	2887	284	38.0	24.5	2.6	0.3	120.59	20.10
PLA/Fe wool 15%	3750	115	60.4	24.6	2.3	1.1	147.64	122.56
PLA/Fe wool 15% after UV	3090	380	46.1	13.7	2.4	0.2	106.61	22.64
PLA/tar 15%	3667	68	81.0	28.6	2.6	1.3	176.04	130.62
PLA/tar 15% after UV	3197	301	51.2	22.5	2.8	0.4	154.11	45.72

**Table 6:** Density measurement values of PLA/wool biocomposite samples and their chemical modifications.

	Dry Weight of Sample, m1 [g]	Wet Sample Weight, m2 [g]	m1/(m1m2) [-]	Density [g/cm^3^]
Pure PLA	1.482	0.275	1.228	1.224
Pure PLA after UV	1.406	0.249	1.215	1.212
PLA/pure wool 15%	1.276	0.205	1.191	1.188
PLA/pure wool 15% after UV	1.34	0.201	1.176	1.173
PLA/acid wool 15%	1.48	0.161	1.122	1.119
PLA/acid wool 15% after UV	1.45	0.188	1.149	1.146
PLA/Fe wool 15%	0.996	0.169	1.204	1.201
PLA/Fe wool 15% after UV	1.4	0.171	1.139	1.136
PLA/tar 15%	1.381	0.27	1.243	1.239
PLA/tar 15% after UV	1.055	0.242	1.298	1.294

## Data Availability

The original contributions presented in this study are included in the article. Further inquiries can be directed to the corresponding author.
